# From Spinal Meningioma to Compressive Hematoma: A Case of Spontaneous Spinal Hematoma Mimicking Meningioma in a Pregnant Woman

**DOI:** 10.7759/cureus.83086

**Published:** 2025-04-27

**Authors:** Yassine Kherchttou, Montacer Ait Riala, Jamal Ouachaou, Hanane Mourouth, Zarrouki Youssef

**Affiliations:** 1 Anesthesia and Intensive Care Unit, Mohammed VI University Hospital, Marrakech, MAR

**Keywords:** acute spinal cord compression, pregnancy, spinal meningioma, spinal mri, spontaneous spinal epidural hematoma

## Abstract

Spontaneous spinal hematomas are rare but potentially life-threatening, particularly during pregnancy, where they can mimic other spinal lesions, complicating diagnosis and management. We report a case of a 26-year-old pregnant woman at 23 weeks of gestation, who presented with sudden-onset paraparesis. Neurological examination revealed signs of spinal cord compression, and MRI suggested a spinal meningioma due to an extramedullary tissue process at the D3-D4 level. Despite a normal coagulation profile, emergency laminectomy (D2-D6) was performed, revealing and evacuating a spontaneous spinal hematoma. No active bleeding source or vascular malformation was identified, and fetal assessments remained stable. The pathophysiology of spontaneous spinal hematomas in pregnancy is multifactorial, involving increased vascular fragility due to pregnancy-induced hemodynamic and hormonal changes, including elevated estrogen and progesterone levels and a hypercoagulable state. Additionally, increased intra-abdominal pressure from the gravid uterus may contribute by exacerbating spinal venous congestion. Distinguishing spinal hematomas from meningiomas is challenging due to overlapping clinical and imaging features; however, the sudden onset of symptoms and characteristic MRI signal patterns can aid in differentiation. This case underscores the importance of considering spontaneous spinal hematoma in pregnant patients with acute neurological symptoms. Early recognition and timely surgical decompression are essential to prevent irreversible neurological deficits and optimize maternal and fetal outcomes. Further research is needed to better understand the underlying mechanisms and improve management strategies for this rare but serious condition.

## Introduction

Spontaneous spinal hematomas (SSHs) are rare but potentially devastating conditions, particularly during pregnancy, where timely diagnosis is critical to prevent permanent neurological damage and fetal compromise. Etiologies include trauma, vascular malformations, anticoagulant therapy, and coagulation disorders, and, less commonly, they occur idiopathically. Pregnancy-related physiological changes (including increased vascular fragility, hypercoagulability, and hemodynamic shifts) heighten the risk of spontaneous bleeding [1].

Due to overlapping clinical and radiological features, SSH may be mistaken for spinal tumors such as meningiomas, which typically have a slower, progressive course. This case describes a pregnant woman with acute paraparesis, initially misdiagnosed with a spinal meningioma based on MRI findings. The case highlights the diagnostic challenges of differentiating spinal hematomas from neoplastic lesions, underscoring the need for prompt recognition and intervention [2].

## Case presentation

A 26-year-old pregnant woman, gravida 4 para 3 (G4P3), at 23 weeks of gestation, presented with a sudden onset of bilateral lower limb weakness associated with severe mid-back pain. She was performing routine household activities at symptom onset and reported no trauma, exertion, or prior neurological complaints. Her medical and surgical history was unremarkable, with no known coagulopathy, and she was not taking any anticoagulants or antiplatelet agents. All previous deliveries were uneventful.

Clinical examination

Neurological examination revealed signs of spinal cord compression, including pain on cervical spine palpation. Reflex testing showed monokinetic osteotendinous reflexes in the upper limbs and diffuse, polykinetic reflexes in the lower limbs. Babinski sign was bilaterally positive, while Hoffmann sign was negative. Motor function was preserved in the upper limbs, but manual muscle testing showed 2/5 strength in the lower limbs. Sensory examination identified a sensitive level at L2, with no coordination disorders noted in the upper limbs. Urinary retention was observed, indicating sphincter involvement.

The case was classified as grade C, according to the Standard Neurological Classification of Spinal Cord Injury by the American Spinal Injury Association (ASIA) classification, with preserved but non-functional motor activity (2/5) below the lesion [3].

Imaging findings

**Figure 1:**
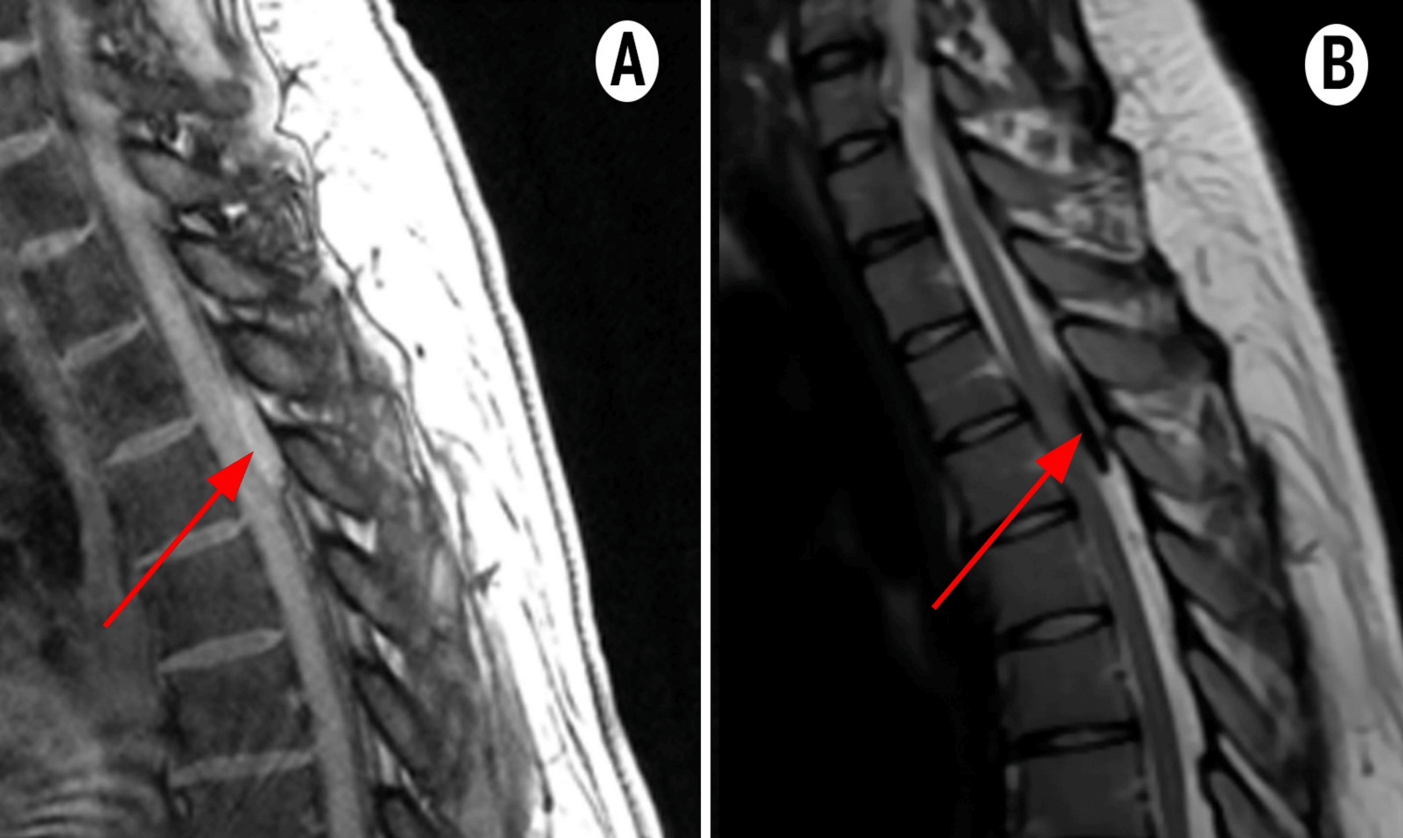
Thoracic spine MRI. Thoracic spine MRI showing an extramedullary tissue process centered on the meninges at the D3-D4 level, measuring 7 × 12 × 28 mm (red arrow). It appears isointense on T1 and T1 fat saturation (A) and hypointense on T2 (B).

Emergency non-contrast MRI (Figure 1) revealed an extramedullary tissue process centered on the meninges at the T3-T4 level, measuring 7 × 12 × 28 mm. The lesion appeared isointense on T1 and T1 fat saturation, hypointense on T2, and isointense on diffusion-weighted imaging. It caused a significant mass effect on the posterior surface of the spinal cord, with intramedullary hypersignal suggestive of spinal cord edema or early ischemic changes. The radiological appearance strongly suggested a spinal meningioma, delaying initial suspicion of hematoma.

Laboratory investigations, including coagulation parameters, were unremarkable, as shown in Table 1.

**Table 1:** Laboratory findings. Hemoglobin, platelets, and renal and liver functions were within normal limits. Coagulation was preserved, with a normal prothrombin ratio and a slightly shortened activated partial thromboplastin time (aPTT), consistent with the hypercoagulable state of pregnancy. C-reactive protein (CRP) was elevated (19.5 mg/dL), likely reflecting a sterile inflammation, as leukocyte count remained normal. Electrolytes were stable, aside from borderline hyperkalemia (4.68 mmol/L), which warranted rechecking. Thyroid function was normal (thyroid-stimulating hormone and T4 were within range), and the lipid profile showed mild hypercholesterolemia and elevated high-density lipoprotein, consistent with physiological changes of pregnancy.

Parameter	Patient value	Reference value
Hemoglobin (g/dL)	13.1	12 – 16
Hematocrit (%)	40.4	37 – 47
Leukocytes (× 10³/uL)	4.46	4 – 10
Neutrophils (× 10³/uL)	2.36	2 – 7.5
Lymphocytes (× 10³/uL)	1.39	1 – 4
Platelets (× 10³/uL)	168	150 – 450
Blood type	O^+^	-
Activated partial thromboplastin time (s)	25.9	30 – 35
Prothrombin ratio (%)	96.8	70 – 100
Urea (mg/dL)	0.27	0.25 – 0.48
Creatinine (mg/dL)	7.2	5 – 9
Sodium (mmol/L)	139	135 – 145
Potassium (mmol/L)	4.68	3.5 – 4.5
C-reactive protein (mg/dL)	19.5	0 – 5
Thyroid-stimulating hormone (mUI/L)	1.97	0.35 – 4.94
Thyroxine (pmol/L)	12.14	9.01 – 19.05
Albumin (g/L)	44.3	32 – 45
Uric acid (mg/L)	44.97	24 – 57
Aspartate transaminase (UI/L)	17	10 – 35
Alanine transaminase (UI/L)	15	7 – 33
Cholesterol (g/L)	2.3	1.54 – 2.01
Triglyceride (g/L)	1.22	0.4 – 1.4
Low-density lipoprotein (g/L)	1.38	0 6 – 1.6
High-density lipoprotein (g/L)	0.68	0.45 – 0.65

Surgical intervention

An emergency laminectomy (D2-D6) was performed to decompress the spinal cord. The patient was positioned in the prone position using a standard Wilson frame to ensure optimal spinal alignment and minimize abdominal compression. Special care was taken to avoid vena cava compression and maintain adequate uteroplacental perfusion. Soft chest and pelvic bolsters were used to offload abdominal pressure, and all pressure points were padded appropriately. Continuous monitoring of maternal hemodynamics and oxygenation was ensured throughout the procedure, as these factors can compromise uteroplacental perfusion, posing risks to both maternal and fetal health.

**Figure 2:**
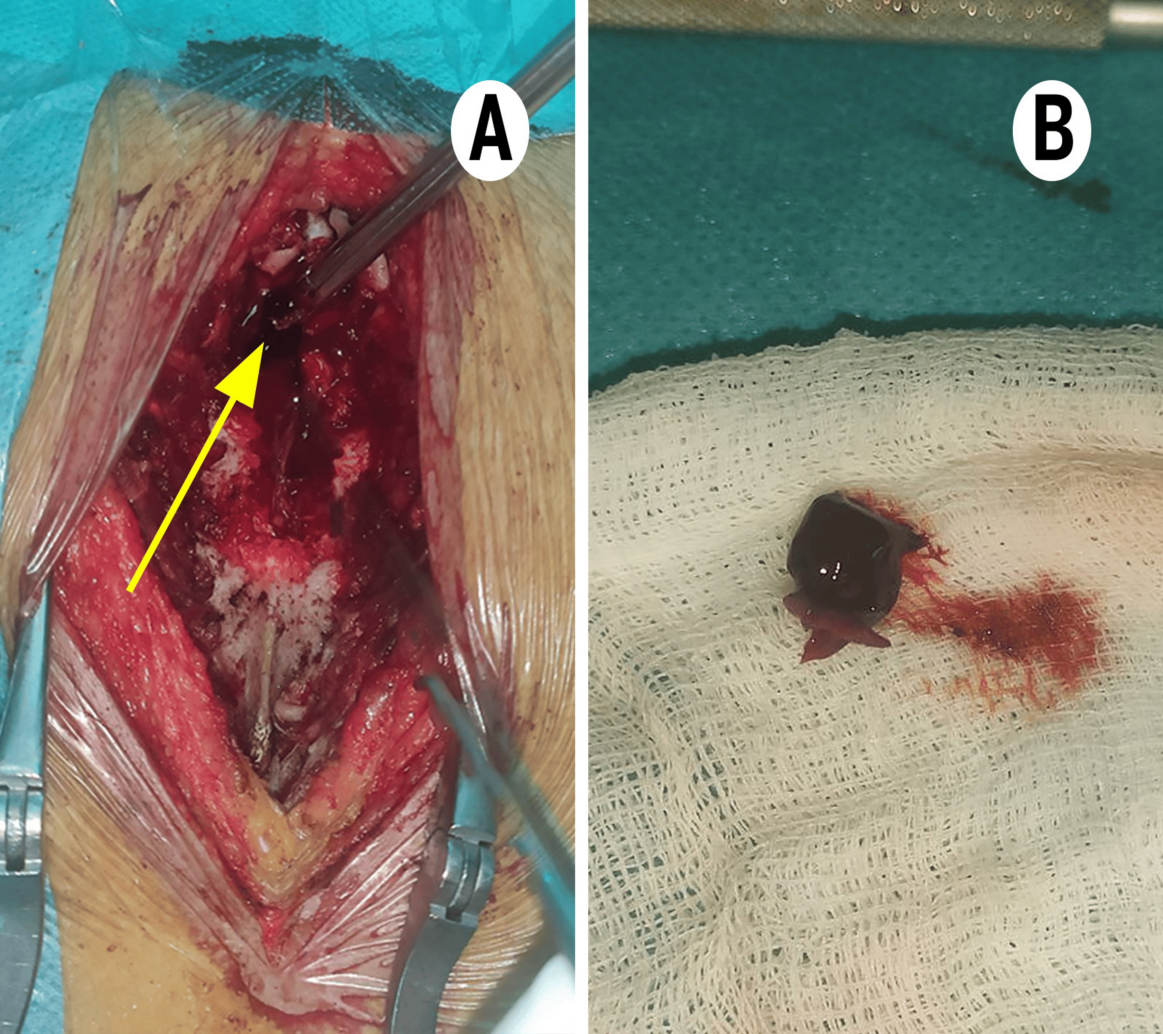
Surgical images. A: Perioperative view showing the compressive spinal hematoma (yellow arrow). B: The spinal compressive hematoma after its extraction.

Intraoperatively, a compressive spinal subdural hematoma was discovered in direct contact with the spinal cord and evacuated, confirming the true etiology (Figure 2). No active bleeding or vascular malformation was identified. Pre- and postoperative fetal evaluations, including Doppler ultrasound and cardiotocography, were unremarkable.

Postoperative course

On postoperative day seven, the patient demonstrated improved strength in proximal lower limb muscles (hip flexion and knee extension at 3/5), while distal motor function remained poor (1/5). Sensory level remained at L2. She tolerated passive mobilization and was referred for intensive multidisciplinary rehabilitation.

Follow-up communication with the patient confirmed progressive motor recovery over the subsequent months. She eventually regained autonomous ambulation and was able to walk without assistance. Furthermore, her pregnancy progressed uneventfully, and she delivered a healthy baby via cesarean section without complications.

## Discussion

Spontaneous spinal hematomas during pregnancy, although rare, pose significant risks due to the potential for rapid neurological deterioration, like all compressive cord injuries. Pregnancy-related vascular changes, including increased blood volume and cardiac output, can lead to increased vascular fragility, making blood vessels more susceptible to rupture. Additionally, hormonal influences, particularly elevated estrogen and progesterone levels, may weaken vascular integrity, further predisposing to hemorrhage [4].

The hypercoagulable state of pregnancy, a physiological adaptation to prevent excessive postpartum hemorrhage, paradoxically increases the risk of spontaneous bleeding [5]. Mechanical factors, such as increased abdominal pressure from the enlarging uterus, can further exacerbate spinal venous congestion, increasing the likelihood of vascular rupture [2].

Spinal meningiomas can occur throughout the spine, but they predominate (90% of cases) in the thoracic region, usually positioned posterolaterally. Most meningiomas are intradural, with only 10% being extradural or dumbbell-shaped [6].

Distinguishing between spontaneous spinal hematomas and spinal meningiomas is particularly challenging due to overlapping clinical and radiological features. Both can present with back pain, radiculopathy, and signs of spinal cord compression. However, the temporal evolution of neurological deficits is a crucial differentiating factor. Spinal meningiomas typically have a slow and insidious onset, with symptoms progressing gradually over weeks to months. This is due to the slow-growing nature of the tumor, allowing for partial adaptation and collateral compensation. In contrast, spontaneous spinal hematomas are characterized by a sudden and rapidly evolving neurological deterioration, often occurring within minutes to hours. This reflects acute compression of the spinal cord by blood accumulation, leaving no time for adaptation, and leading to abrupt motor and sensory deficits. In our case, the acute paraplegia in a previously healthy woman strongly favored a hemorrhagic etiology, despite initial MRI findings suggestive of a tumor [7,8].

MRI is the primary imaging modality used for evaluating spinal lesions, but both hematomas and meningiomas appear as space-occupying masses compressing the spinal cord, complicating differential diagnosis. However, spinal hematomas, particularly in the acute phase, typically appear hyperintense on T1-weighted images and hypointense on T2-weighted images, with varying signal intensities depending on blood degradation. In contrast, meningiomas are typically round, broad-based masses with a “tail” extending into the dura, and 5% of cases show calcifications. Radiological characteristics are listed in Table 2 [6].

**Table 2:** Radiological characteristics of spinal hematomas and spinal meningiomas.

Feature	Spinal hematoma	Spinal meningioma
T1-weighted MRI	Hyperintense (acute phase)	Isointense or hypointense
T2-weighted MRI	Hypointense (acute), hyperintense (subacute/chronic)	Isointense or hyperintense
Location	Variable, epidural/subdural possible	Dural-based, often with a dural “tail” sign
Other clues	Can have mixed signal intensities due to blood degradation	May have calcifications (5%)

Given the potential for rapid neurological deterioration, timely diagnosis and intervention are critical. In cases of spinal hematoma, urgent surgical decompression, as performed in our patient, is often required to prevent irreversible spinal cord injury [9]. Understanding these differences is crucial for accurate diagnosis and appropriate management, especially in pregnant patients, where both maternal and fetal outcomes must be considered.

Additionally, the prone position, commonly used for spinal surgeries, poses specific risks for pregnant patients, particularly during the second and third trimesters, due to physiological changes such as increased uterine size, elevated intra-abdominal pressure, and aortocaval compression. These changes can lead to hemodynamic instability, reduced venous return, impaired respiratory mechanics, and potential fetal compromise if not adequately addressed.

Therefore, careful anesthetic management and positioning techniques, including appropriate padding, uterine displacement, and continuous maternal-fetal monitoring, are essential to minimize these risks and ensure both maternal and fetal safety during surgery [10].

## Conclusions

This case highlights the diagnostic challenge of differentiating spinal hematoma from spinal meningioma, particularly in pregnancy. The potential for rapid neurological deterioration necessitates early recognition and intervention to prevent permanent deficits.

Clinicians should maintain a high index of suspicion for spontaneous spinal hematoma in pregnant patients with acute neurological symptoms, especially when imaging findings are inconclusive. Timely decompression surgery, coupled with careful anesthetic management, remains the cornerstone of optimizing both maternal and fetal outcomes.
